# Abstract of Proceedings of the Buffalo Medical Association

**Published:** 1867-12

**Authors:** T. M. Johnson

**Affiliations:** Sec’y.


ART. IV—*Abstract of Proceedings of the Buffalo Medical Association.*

TUESDAY EVENING, November 5th, 1867.

The meeting was called to order by the President. Members present—Drs. Eastman, Jansen, Abbott, L. F. Harvey, C. F. A. Nichell, Wetmore, Strong, Daggett, Gleason, Rochester, Congar, Smith, Potter and Johnson.

The report of the proceedings of the last meeting were read and approved.

DR. ROCHESTER moved that Dr. Eddy, late of Lewiston, now of

Buffalo, be invited to participate in the proceedings of the meeting. Carried.

The Secretary read a communication from the Business Committee of the Young Men's Association, asking this Association to pay the pro rata expense of the janitor.

Dr. Rochester moved that the communication be received, and that if the Young Men's Association will guarantee the proper care of the rooms, this Association be directed to pay the amount required by the Young Men's Association. After brief informal discussion the motion was carried.

The President announced that the consideration of the revised Constitution and By-Laws was the next business in order.

Dr. Strong moved that the Secretary read the Constitution and By-Laws as reported by the Committee, and unless objection be made to any part thereof, the whole be adopted as read. The motion was carried.

Dr. Rochester moved that Article II. of Chapter VI. which relates to impeachment, be so modified as to direct that when charges of impeachment are made against a member, the case shall be investigated by a committee before coming before the Association. Carried.

Dr. Strong moved that the consideration of the fee bill be deferred until the next regular meeting. Carried.

Dr. C. F. A. Nichell said, that Dr. Miner being unable to be present had invited him to present two specimens of disease of nerve, of rare interest and value. The first is a section of the median nerve, showing in its centre an enlarged condition, the result of a gun-shot wound. The patient, W. C., accidentally wounded himself in October, 1866, the ball passing from within outward, two inches above the condyles of the humerus. Nine weeks from the time of receipt of the injury the wound had closed, but the patient suffered the most excrutiating pain in the hand, which was flexed. At the expiration of twenty-two weeks he presented himself to Dr. Miner for treatment, the pain being so intense as to make existence intolerable, and calling for relief by some means, even amputation, if necessary. He was much emaciated, and the digestive functions impaired by the pain and the large amount of opium taken. An incision was made upon the inner margin of the biceps muscle, four or five inches in extent, readily exposing the median nerve in its course over the brachial artery. The nerve was found closely involved in the cicatrix of the wound, and was with some difficulty dissected out; the surrounding tissues were carefully separated, and three inches of the nerve removed. The long confinement of the fingers, and inflammation of the tissues of the joint, had caused false anchylosis, which was broken up with considerable force, giving way with a snap, similar to that of the fracture of a bone. The wound, mainly, united by first intention, the ligatures separating on the seventh day. Artificial motion was employed to restore the mobility of the fingers, which, at the expiration of ten months, is nearly as perfect as ever. Sensation, except to a certain extent along the anterior surface of the forearm, has not been impaired, and the patient again attends to his business.

Dr. Miner has examined the statistics of the Crimean war, and finds but twenty-three cases of gun-shot injury to the larger nerves, such as the sciatic, median, and ulnar, are reported, of which nine, or forty-one per cent. proved fatal, five dying from tetanus. The U. S. Army Medical Museum contains but two specimens of exsected nerves, both instances followed by very partial relief from neuralgic pain.

The second case is that of Mr. M., aged 35 years, who presented himself, a few days since, for the removal of a tumor occupying the lower fourth of the left arm, internal to the biceps muscle. The growth measured about three inches in length and two in breadth, being elastic to the touch, and presenting the characteristics of an encysted tumor. Its history extended over a period of five years, growing slowly, and at no time interfering with the functions of the arm, or causing very great inconvenience, until within the last two months, when it rapidly augmented in size and became exceedingly tender on pressure, and too painful for endurance. An incision of three inches over the tumor having been made, and the tissues pressed one side, the neuromatous condition of the median nerve was discovered, about one-third of the fibres of which were widely separated and expanded over the anterior surface, again uniting with the common trunk just above the bend of the elbow-joint. Upon its posterior surface the nerve fibres were more closely connected with each other, and not so intimately incorporated with the cyst, their separation from the same being readily effected. The two extremities of the nerve having been divided, the tumor was easily removed, there being no more adhesions than are ordinarily found in encysted growths.

Partial paralysis of the fore-arm and hand followed the operation, but all neuralgic pains ceased. One month from the operation there is partial restoration of the functions of both hand and arm, the remaining disability existing mainly in the loss of the power of the extensor muscles of the hand, while the flexors appear nearly perfect. The fact that so large a nerve can be removed with so little loss of sensation and motion, Dr. Miner regards as of more interest, perhaps, than any other in the cases presented, and on this account, as well as from the rarity of tumor of nerve, and the perfect success of the treatment in relieving pain, has invited me to present them.

Dr. Eastman remarked, that he fully concurred with the views advanced in the cases just reported. Dr. E. related a similar case of tumor upon the foot of a lady. The tumor was removed with almost entire relief to the patient.

Dr. Rochester said, that he might not be present at the next meeting, when the fee bill would probably be acted upon, and would therefore like to call the attention of the members present to the subject of examinations for life insurance. He thought it was well worth five dollars to make such examinations. Had seen articles in medical journals setting forth the necessary care and time requisite to a proper examination and advocating a higher rate of compensation. He arose more especially to speak of the custom now prevailing with physicians of giving certificates to life insurance agents in regard to the physical condition of their patients with reference to life insurance. He had often made such certificates and always charged for them. If our opinion is worth anything, it is worth paying for. It is entirely to the interest of the company to have the opinion of the family physician, and the company should pay for it.

Scarlatina was reported as prevailing to considerable extent.

Adjourned. T. M. Johnson, M. D., Sec'y.

